# Metabolic pathways and cell death modalities in diabetic complications: unraveling pyroptosis, ferroptosis, cuproptosis, and disulfidptosis

**DOI:** 10.1038/s41420-026-03162-3

**Published:** 2026-05-22

**Authors:** Zifan Tian, Yulong Cao, Jiaheng Liu, Xuehong Zheng, Jiaxu Li, Jingyang Zhao, Panpan Xia, Deju Zhang, Xiao Liu, Jianping Liu, Jing Zhang, Peng Yu, Wenting Wang

**Affiliations:** 1 Department of Endocrinology and Metabolism, The Second Affiliated Hospital, Jiangxi Medical College, Nanchang University, Nanchang, Jiangxi China; 2 Queen Mary College of Nanchang University, Nanchang, Jiangxi China; 3 Institute for the Study of Endocrinology and Metabolism in Jiangxi Province, Nanchang, China; 4 Eurofins BioDiagnostics Inc., River Falls, WI USA; 5 Department of Cardiology, Sun Yat-sen Memorial Hospital of Sun Yat-sen University, Guangzhou, Guangdong China; 6 Department of Anesthesiology, The Second Affiliated Hospital, Jiangxi Medical College, Nanchang University, Jiangxi Nanchang, China; 7 Department of Anesthesiology, The Second Affiliated Hospital of Hainan Medical University, Haikou, Hainan China

**Keywords:** Apoptosis, Metabolic disorders

## Abstract

The pathogenesis of diabetic complications involves a complex interplay of metabolic disturbances, in which diverse programmed cell death (PCD) pathways play a pivotal role. This article systematically reviews the molecular mechanisms of four novel types of metabolic cell death—pyroptosis, ferroptosis, cuproptosis and disulfidptosis—with a focus on their specific pathological roles in diabetic kidney disease, retinopathy, cardiovascular complications and neuropathy. It also analyzes the crosstalk and synergistic regulatory networks among these four cell death modalities. Studies have demonstrated that in the diabetic microenvironment, these four PCD pathways do not act independently; instead, they form an interconnected regulatory network centered on oxidative stress, inflammatory signaling and metal ion homeostasis imbalance. This network collectively amplifies metabolic stress, inflammatory responses and oxidative damage, thereby accelerating the progression of multi-organ injury in diabetes. Furthermore, this article explores the therapeutic implications of this network-based perspective, highlighting the potential value of multi-target intervention strategies in the treatment of diabetic complications. In summary, this review clarifies the intricate associations between metabolic cell death pathways and diabetic complications, and underscores the importance of the crosstalk among different cell death modalities. It thus provides a novel theoretical framework for understanding the systemic pathophysiological mechanisms of diabetes, and offers insights and directions for the development of novel combination therapeutic strategies.

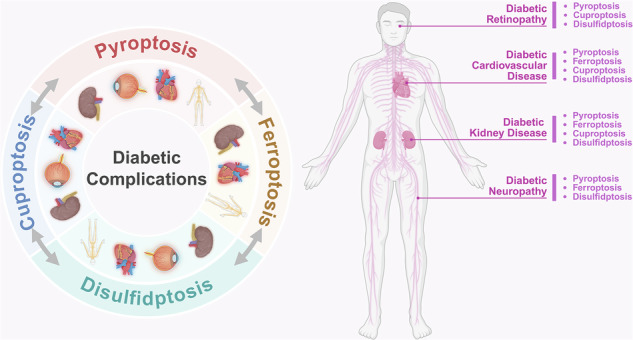

## FACTS

- Pyroptosis and ferroptosis can achieve synergistic amplification via a dual positive feedback pathway involving the “oxidative stress-inflammatory signaling interaction.”
- Through “metal ion metabolic coupling,” cuproptosis and ferroptosis can mutually exacerbate each other.
- Pyroptosis and cuproptosis are interconnected through a three-tiered interactive pathway: “Copper metabolism disorder — inflammatory signaling activation — mitochondrial damage.”
- Disulfidptosis may serve as an interactive hub that links “metabolism-oxidative stress-cytoskeleton” and is associated with pyroptosis, ferroptosis, and cuproptosis.
- A combined regimen of “cytokine inhibitors + antioxidants” has been designed as a clinical protective strategy against diabetic complications based on the interactive mechanisms of four types of cell death.

## Introduction

The International Diabetes Federation (IDF) reports in the 11th edition of the IDF Diabetes Atlas (2025) that 589 million adults (aged 20 to 79) worldwide have diabetes [1]. This figure is expected to increase to 853 million by 2050. In 2024, diabetes was responsible for 3.4 million deaths and resulted in at least USD 1 trillion in healthcare expenditure, marking a 338% increase over the past 17 years. Chronic hyperglycemia caused by diabetes is one of the most serious public health challenges. It can lead to microvascular and macrovascular complications, such as kidney disease, retinopathy, neuropathy and cardiovascular disease. These can ultimately result in severe disability and death. Although glucose- and lipid-lowering therapies can slow the progression of the disease, the burden of diabetes-related complications remains significant. The IDF notes that people with type 2 diabetes have a 73% higher risk of myocardial infarction, a 54% higher risk of stroke, and an 84% higher risk of heart failure [1]. Furthermore, multinational epidemiological studies reveal that the prevalence of diabetic kidney disease (DKD) among people with type 2 diabetes in Malaysia is 37.5%; the prevalence of diabetic peripheral neuropathy (DPN) among people with diabetes in Pakistan is 33.7%; and the prevalence of diabetic retinopathy (DR) among adults with type 2 diabetes in the Adama region of Ethiopia is 34.6% [2–4].

In recent years, metabolic cell death, programmed cell death caused by disruption of specific metabolic pathways, has been increasingly recognized as a contributor to the development of diabetic complications and a potential entry point to overcome therapeutic barriers [5, 6]. The major types currently highlighted include pyroptosis, ferroptosis, cuproptosis, and disulfidptosis. Pyroptosis is a lytic cell death triggered by inflammasome activation and mediated by gasdermin proteins, accompanied by the release of inflammatory cytokines such as IL-1β and IL-18 [7]. Ferroptosis is an iron-dependent cell death driven by lipid peroxidation and regulated by redox systems such as GPX4 and SLC7A11 [8]. Cuproptosis occurs with copper overload, leading to mitochondrial protein lipoylation and loss of Fe–S clusters, culminating in metabolic collapse [9]. Disulfidptosis is triggered by NADPH depletion, resulting in abnormal disulfide accumulation and cytoskeletal breakdown [10]. Importantly, these death programs are not isolated; within the diabetic microenvironment, they may interact dynamically and jointly amplify inflammation and oxidative damage, yet comprehensive documentation of their synergistic interactions and translational activity across more than one death mode remains insufficient.

This review summarizes the molecular mechanisms and pathological roles of pyroptosis, ferroptosis, cuproptosis and disulfidptosis in diabetic complications, exploring potential avenues such as multi-target combination therapy, biomarker discovery and organoid model development. The review reveals that oxidative stress plays a common triggering and amplifying role in pyroptosis, ferroptosis, cuproptosis and disulfidptosis. Inflammatory signaling, particularly the NLRP3/NF-κB pathway, interlinks pyroptosis with other forms of cell death. Meanwhile, imbalances in metal ion homeostasis, such as those involving iron and copper, are closely linked to overall metabolic dysregulation in diabetes and to ferroptosis and cuproptosis. By examining the ‘oxidative stress-inflammation-metal ions’ metabolic core axis, we can systematically elucidate the synergistic network among these four forms of cell death. This provides a wholly novel approach to understanding the systemic pathophysiological mechanisms underlying diabetic complications.

## Physiological and pathological functions of metabolic cell death

A comprehensive understanding of PCD is essential for interpreting diabetic tissue injury. Four major PCD modalities—pyroptosis, ferroptosis, cuproptosis, and disulfidptosis—have emerged as central contributors in diabetes and its complications. Each modality is triggered by distinct metabolic or stress cues but often converges on shared nodes of oxidative stress, redox imbalance, and mitochondrial dysfunction.

Pyroptosis is an inflammatory programmed cell death characterized by cell swelling, membrane permeabilization, and lytic rupture accompanied by the release of IL-1β and IL-18 [11, 12]. The canonical pathway involves inflammasome assembly, including NLRP3, ASC, and pro-caspase-1, leading to caspase-1 activation and cleavage of gasdermin D (GSDMD). The GSDMD N-terminal fragment inserts into the plasma membrane, forming pores that drive osmotic lysis and inflammatory cytokine release [13, 14]. Non-canonical pyroptotic pathways involve caspase-4/5 in humans and caspase-11 in mice, which directly activate GSDMD, and other gasdermins such as GSDME may execute similar pore-forming functions [15, 16]. Hyperglycemia-induced reactive oxygen species (ROS) activate TXNIP, which in turn promotes NLRP3 inflammasome assembly, linking metabolic stress to pyroptotic cell death [17]. This pathway is widely implicated across renal, vascular, and neural tissues under diabetic conditions.

Ferroptosis is an iron-dependent form of cell death driven by lipid peroxidation and characterized by the accumulation of lipid hydroperoxides and ROS [18]. It is tightly regulated by the system Xc⁻–GSH–GPX4 axis, where SLC7A11 mediates cystine uptake to support glutathione (GSH) synthesis, and GPX4 reduces lipid peroxides to maintain membrane integrity [19–23]. Pharmacologic inhibition of SLC7A11 (e.g., erastin) or GPX4 (e.g., RSL3) triggers ferroptosis, while FSP1, Trim21, and FTH1 modulate sensitivity through iron homeostasis and autophagic regulation [24–26]. ACSL4 promotes ferroptosis by incorporating polyunsaturated fatty acids into membrane phospholipids, increasing susceptibility to peroxidation [27, 28]. Ferroptosis has been observed in multiple diabetic tissues, including the kidney, heart, and peripheral nerves, highlighting its broad pathological relevance.

Cuproptosis is a recently identified form of PCD induced by intracellular copper overload [29]. It differs mechanistically from pyroptosis and ferroptosis, occurring through the binding of copper to lipoylated TCA cycle proteins such as DLAT, DLST, and LIAS, which destabilizes iron–sulfur cluster proteins and triggers proteotoxic stress and mitochondrial dysfunction [30]. FDX1 reduces Cu²⁺ to the more reactive Cu⁺ and facilitates lipoylation, amplifying copper-mediated cytotoxicity [31]. Cellular sensitivity to cuproptosis is modulated by copper transporters SLC31A1 and ATP7B, and can be attenuated by glutathione-mediated copper chelation [32, 33]. Although less studied in diabetes than pyroptosis and ferroptosis, cuproptosis may contribute to mitochondrial metabolic stress under hyperglycemic conditions.

Disulfidptosis arises from NADPH depletion and excessive disulfide bond accumulation [34]. This leads to aggregation of disulfide-rich cytoskeletal proteins (ACTB, MYH9, FLNA, FLNB) and collapse of the F-actin network, resulting in structural failure and cell death [35]. Disulfidptosis is initiated via SLC7A11-mediated cystine uptake; under glucose restriction or impaired pentose phosphate pathway (PPP) activity, NADPH becomes insufficient to reduce imported cystine, producing disulfide stress and catastrophic protein crosslinking [36, 37]. The RAC1–WAVE regulatory complex (WRC), normally promoting actin polymerization, may further exacerbate cytoskeletal disintegration under these altered redox conditions [38] (Table 1).

**Table 1:** Comparison of four types of cell death.

Cell Death Type	Core Triggering Factors	Key Molecular Markers	Potential Therapeutic Targets	References
Pyroptosis	High glucose-induced ROS; mtDNA release; Hyperglycemia-induced activation of NLRP3 inflammasome	IL-1β; IL-18; NLRP3; ASC; pro-caspase-1/caspase-1; GSDMD; GSDME	Inflammasome inhibitors; Gasdermin blockers;	[11–17]
Ferroptosis	Iron overload; Lipid peroxidation; Redox imbalance (GSH depletion, GPX4 inactivation)	GPX4; SLC7A11; GSH; NRF2; FTH1; TFR1; 4-HNE; MDA; ROS	GPX4 activators; Iron chelators; Lipid peroxidation inhibitors; AMPK/NRF2 activator; Nrf2/HO-1 activator	[18–28]
Cuproptosis	Intracellular copper overload; Impaired copper homeostasis Epigenetic dysregulation	SLC31A1; ATP7A; ATP7B; ATOX1; FDX1	Copper chelators; Protein stabilizers; Metal ion regulators	[29–33]
Disulfidptosis	NADPH depletion; Disulfide stress; SLC7A11 overexpression	SLC7A11; NADPH; GSH; TXNIP;	SLC7A11 inhibitors; NADPH precursors; Redox modulators; Cytoskeletal protectors	[34–38]

## The role of pyroptosis in diabetes and its complications

### Pyroptosis in diabetic kidney disease

DKD is a major microvascular complication of diabetes, characterized by progressive glomerulosclerosis, podocyte loss, and interstitial fibrosis [39, 40]. In the hyperglycemic inflammatory milieu, pyroptosis has been established as a driver of renal injury, largely through inflammasome activation and GSDMD cleavage in renal parenchymal and immune cells, with consequent IL-1β/IL-18 release that sustains inflammation and promotes fibrotic remodeling [41–43].

Mechanistically, upstream metabolic cues can directly couple glucose handling to pyroptotic signaling. In tubular epithelial cells, the SGLT2–SGK1 axis provides a diabetes-relevant route linking tubular glucose reabsorption to NLRP3 activation and inflammatory cell death [44]. In parallel, oxidative stress amplification—classically through ROS-driven TXNIP signaling—acts as a reinforcing loop that lowers the threshold for inflammasome activation in DKD contexts [45]. Collectively, these pathways position pyroptosis not merely as a downstream consequence of injury but as a feed-forward mechanism that accelerates renal inflammation and fibrosis in DKD.

### Pyroptosis in diabetic retinopathy

In DR, hyperglycemia induces endothelial dysfunction and chronic inflammation, and pyroptosis has emerged as a mechanistic contributor to retinal injury. Canonically, high glucose can activate ATP–P2X7 receptor signaling to promote inflammasome activation, caspase-1 cleavage, and GSDMD-mediated pore formation in retinal endothelial cells [46].

Beyond this canonical route, transcriptomic and experimental validation studies in DR samples/cell models report upregulation of key pyroptosis-associated genes (including inflammasome/caspase components and gasdermins), supporting a broader activation landscape rather than a single linear pathway [47]. Non-canonical execution has also been described via the caspase-3/GSDME axis, extending pyroptotic-like membrane permeabilization to contexts traditionally associated with apoptosis; TNFSF15 has been proposed to restrain this process by interacting with GSDME and limiting pore formation [48, 49]. Together, these findings suggest DR-associated pyroptosis is organized around a limited set of executioner nodes (gasdermins/caspases) but can be initiated by multiple upstream stressors in the diabetic retina.

### Pyroptosis in diabetic cardiovascular complications

Diabetic cardiovascular complications (DCVC) are leading causes of morbidity and mortality in diabetes, encompassing phenotypes such as diabetic cardiomyopathy and ischemia vulnerability [50]. Pyroptosis contributes to both myocardial injury and vascular dysfunction through convergent inflammasome-centered mechanisms [51].

At the transcriptional level, analysis of transcriptional regulation revealed that METTL14 was significantly downregulated in the high-glucose model [52]. Overall, m6A levels in cardiac muscle were reduced and TINCR levels increased. TINCR directly bound to and stabilized NLRP3 mRNA, thereby enhancing its expression and triggering pyroptosis in cardiac muscle. Mitochondrial stress is another major convergence point. Lipotoxicity-induced FIS1 upregulation exacerbates mitochondrial fragmentation and increases mitochondrial ROS, which promotes NLRP3-dependent pyroptosis in diabetic cardiomyopathy [53]. In addition, mitochondrial damage with mtDNA leakage can activate the cytosolic DNA sensor cGAS–STING pathway, further promoting cardiac pyroptosis and hypertrophic remodeling in diabetic models [54]. Consistent with this “mitochondria to inflammasome” logic, protective strategies that interfere with inflammasome assembly have shown benefit; for example, GDF11 inhibits inflammasome activation by targeting ASC and attenuates high glucose–induced cardiomyocyte pyroptosis [55].

### The mechanism of pyroptosis in diabetic neuropathy

DN involves chronic metabolic stress and neuroinflammation, and pyroptosis-related pathways have been linked to both pain sensitization and tissue injury [56, 57]. In painful diabetic neuropathy (PDN), TANK-binding kinase 1 (TBK1) activation in spinal dorsal horn microglia promotes pyroptosis through noncanonical NF-κB pathway activation and subsequent NLRP3 inflammasome assembly [58]. The P2X7R/NLRP3 signaling axis similarly facilitates neuroinflammation in diabetic peripheral neuropathy, where Tangzu granule treatment demonstrates therapeutic potential by suppressing this pathway and reducing caspase-1 activation, GSDMD cleavage, and IL-1β/IL-18 release [59]. Complementing these findings, reactive oxygen species have been identified as crucial inducers of neurological damage through pyroptosis mechanisms, with VEGF-loaded ROS-responsive nanodots showing promise in ameliorating diabetic peripheral neuropathy by mitigating this oxidative stress-induced cell death [60].

**Fig. 1:**
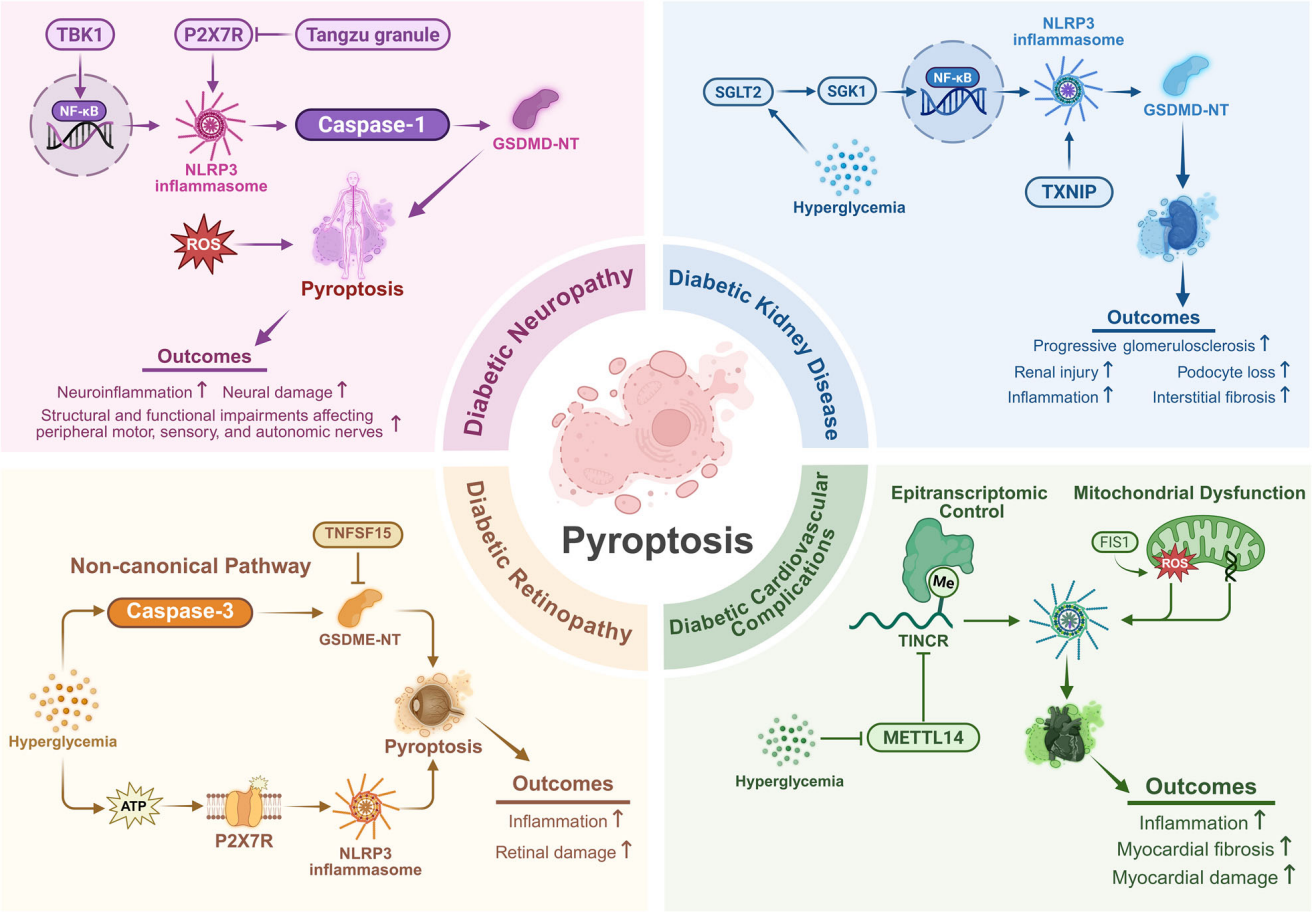
Metabolic dysregulation and pyroptosis in diabetic complications. In Diabetic Kidney Disease (DKD) (top right), hyperglycemia initiates pyroptosis via the SGLT2-SGK1-NF-κB signaling axis alongside TXNIP-mediated NLRP3 inflammasome activation. In Diabetic Neuropathy (DN) (top left), pyroptosis is driven by TBK1-mediated NF-κB activation and P2X7R-dependent NLRP3 inflammasome assembly. This cascade can be pharmacologically modulated by agents such as Tangzu granule. In Diabetic Retinopathy (DR) (bottom left), alongside canonical ATP/P2X7R/NLRP3 signaling, a non-canonical pathway is highlighted where Caspase-3 cleaves GSDME to generate the pore-forming GSDME-NT fragment, a process negatively regulated by TNFSF15. In Diabetic Cardiovascular Complications (DCVC) (bottom right), myocardial pyroptosis is triggered by mitochondrial dysfunction-induced inflammasome activation and is concurrently exacerbated by epitranscriptomic regulation via the METTL14-TINCR-NLRP3 axis. DKD: Diabetic Kidney Disease; SGLT2: Sodium-Glucose Cotransporter 2; SGK1: Serum- and Glucocorticoid-Inducible Kinase 1; NF-κB: Nuclear Factor Kappa B; TXNIP: Thioredoxin-Interacting Protein; NLRP3: NOD-like receptor family pyrin domain-containing 3; DN: Diabetic Neuropathy; TBK1: TANK-Binding Kinase 1; P2X7R: P2X Purinoceptor 7 Receptor; DR: Diabetic Retinopathy; Caspase-3: Cysteine-Aspartic Acid Protease 3; GSDME: Gasdermin E; GSDME-NT: Gasdermin E N-Terminal Fragment; TNFSF15: Tumor Necrosis Factor Superfamily Member 15; DCVC: Diabetic Cardiovascular Complications; METTL14: Methyltransferase Like 14; TINCR: Tissue Differentiation Inducing Non-Protein Coding RNA.

These findings collectively establish pyroptosis as a critical pathological mechanism across the spectrum of diabetic neuropathic complications, revealing multiple cell type regulatory networks and offering promising targets for therapeutic intervention in these challenging conditions (Fig. 1).

## Role of ferroptosis in diabetes and its complications

### Role of ferroptosis in diabetic neuropathy

In the context of peripheral neuropathy, Honokiol has been demonstrated to attenuate high glucose-induced ferroptosis in Schwann cells through activation of the AMPK/SIRT1/PGC-1α pathway, revealing a potential therapeutic avenue for intervention [61]. Particularly noteworthy is ALOX15, which has been identified as a central hub gene functionally connecting DPNP with ferroptosis execution; mechanistic studies confirm that its targeted knockdown effectively alleviates high glucose-induced ferroptosis through substantial reduction of lipid peroxidation and intracellular iron accumulation [62].

### Ferroptosis in diabetic cardiovascular disease

In DCVC, ferroptosis has been reported as a contributor to cardiomyocyte injury and cardiac microvascular dysfunction, particularly under conditions of heightened oxidative stress and mitochondrial disturbance [63].

NRF2-centered antioxidant signaling repeatedly emerges as a protective node; sulforaphane has been described to prevent ferroptosis through AMPK/NRF2-related activation of endogenous antioxidant defenses [64]. In addition, inhibition of CAV1 has been reported to attenuate diabetic cardiomyopathy by reducing ferroptosis via NRF2-associated glutathione metabolism support, reinforcing NRF2 as a convergence point for anti-ferroptotic protection [65]. Beyond transcriptional antioxidant control, mitochondrial quality control and lipid remodeling pathways also shape ferroptotic sensitivity. Nicorandil has been reported to alleviate cardiac microvascular ferroptosis through a mitochondria-localized AMPK–Parkin axis coupled to ACSL4 regulation, linking mitochondrial homeostasis with suppression of lipid peroxidation–prone membrane remodeling in diabetic cardiovascular tissues [66].

### Ferroptosis in diabetic kidney disease

**Fig. 2:**
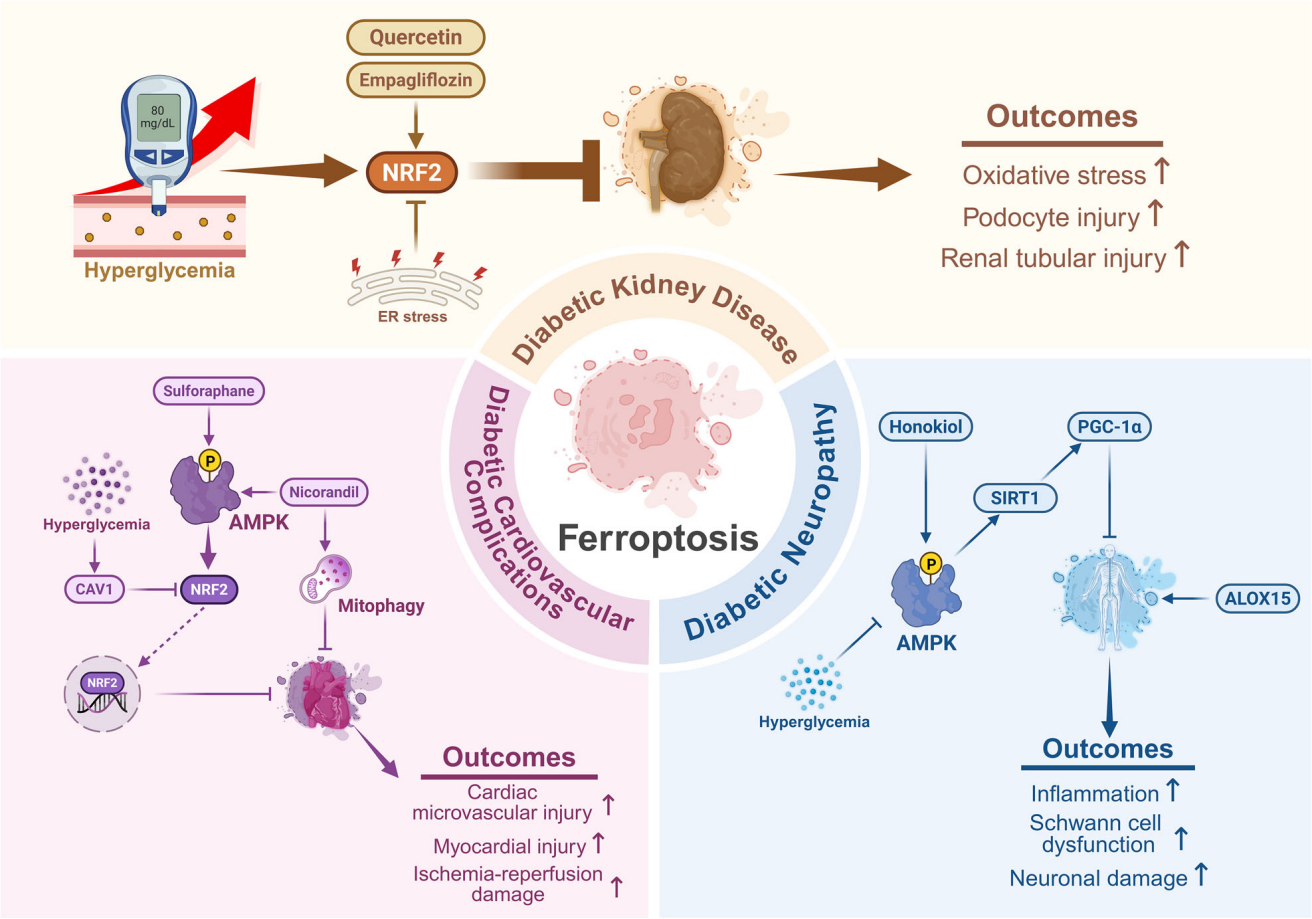
Metabolic dysregulation and ferroptosis in diabetic complications. In Diabetic Kidney Disease (DKD) (top), ferroptosis is prominently regulated by the NRF2 antioxidant signaling axis (targeted by Empagliflozin and Quercetin) and Endoplasmic Reticulum Stress (ERS) responses. In Diabetic Neuropathy (DN) (bottom right), ferroptosis-mediated neuronal injury is governed by the AMPK/SIRT1/PGC-1α axis, which is activated by Honokiol. A pro-ferroptotic enzyme ALOX15 is also shown to induce the ferroptotic process. In Diabetic Cardiovascular Complications (DCVC) (left), ferroptotic injury is primarily mediated by the AMPK/NRF2 pathway. Pharmacological agents such as Sulforaphane and Nicorandil enhance this protective signaling. Notably, Nicorandil concurrently suppresses ferroptosis by promoting mitophagy. Conversely, upregulated CAV1 exacerbates ferroptotic myocardial damage by inhibiting NRF2 activation. DKD: Diabetic Kidney Disease; NRF2: Nuclear Factor Erythroid 2-Related Factor 2; ERS: Endoplasmic Reticulum Stress; DN: Diabetic Neuropathy; AMPK: Adenosine Monophosphate-Activated Protein Kinase; SIRT1: Sirtuin 1; PGC-1α: Peroxisome Proliferator-Activated Receptor Gamma Coactivator 1α; ALOX15: Arachidonate 15-Lipoxygenase; DCVC: Diabetic Cardiovascular Complications; CAV1: Caveolin 1.

In DKD, ferroptosis has been linked to renal tubular and podocyte injury and may act alongside inflammation and fibrosis to amplify functional decline [63, 67]. A consistent protective theme is restoration of NRF2-associated antioxidant capacity and reinforcement of GPX4/SLC7A11-related defenses. Empagliflozin has been reported to attenuate renal tubular ferroptosis via AMPK/NRF2 activation, accompanied by upregulation of GPX4, FTH1, and SLC7A11, which together support lipid peroxide detoxification and iron buffering [68]. Quercetin has likewise been described to protect against DKD-associated ferroptosis through NRF2/HO-1-related antioxidative programming [69]. Cellular stress states can further tune ferroptosis vulnerability in DKD. ER stress has been linked to ferroptotic regulation through the XBP1–Hrd1–Nrf2 axis, providing a mechanistic bridge between stress-response remodeling and iron-dependent lipid injury in diabetic kidneys [70]. Overall, DKD-associated ferroptosis appears to be governed by a limited set of convergent control nodes, with NRF2-related antioxidant defense acting as a core restraint while metabolic and stress-response pathways modulate the threshold for injury progression (Fig. 2).

## Role of cuproptosis in diabetes mellitus and its complications

### Mechanism of cuproptosis in diabetic kidney disease

In DKD, it is the systemic metabolic disturbances caused by diabetes that initiate cuproptosis. The renal copper levels of STZ-induced diabetic rats were significantly higher than those of normal control rats [71]. This accumulation is driven by a combination of factors, including impaired copper excretion due to tubular degeneration, as well as reduced levels of zinc and magnesium in the kidneys, which weaken the function of the antioxidant enzymes involved in copper metabolism [72]. Under high-glucose conditions, the expression of the copper transporter CTR1 increases, resulting in the accumulation of copper within cells [73]. The Cu²⁺ is then transported into the mitochondrial matrix, where it is reduced to Cu⁺ by FDX1 [74]. Reactive Cu⁺ then binds to lipoylated TCA enzymes (DLATs), triggering the aggregation and disruption of iron-sulfur clusters. This subsequently leads to mitochondrial collapse and cuproptosis. Ultimately, cuproptosis targets renal tubular epithelial cells and glomerular mesangial cells, leading to their degeneration and apoptosis. This results in pathological changes in diabetic rat models, such as glomerular hypertrophy, thickening of the basement membrane, and tubular dilation and degeneration. Furthermore, a previous study investigated the effects of ACEI (perindopril) and ARB (valsartan) on the levels of zinc, magnesium, copper and iron in the kidneys of diabetic rats, and how these effects can reduce oxidative stress and delay the progression of DKD [71]. Trientine is a highly selective copper chelator that reduces kidney damage in DKD by removing excess free copper [75]. This is achieved by reducing urinary albumin excretion and inhibiting renal fibrosis. However, most existing studies still focus on omics analysis. Future research should investigate the underlying mechanisms further through experimental validation.

### Mechanism of cuproptosis in diabetic retinopathy

Although there is currently no direct evidence linking cuproptosis to DR, clinical and experimental studies suggest that disturbances in copper homeostasis may contribute to DR pathogenesis. Data from the National Health and Nutrition Examination Survey (NHANES) reveal a U-shaped relationship between copper intake and DR risk, with both insufficient and excessive intake increasing the risk of the disease (multivariable adjusted OR = 0.48, 95% CI = 0.30–0.77 for optimal copper intake) [76].

At a molecular level, an excess of copper binds directly to the lipoylated components of the tricarboxylic acid cycle, particularly within metabolically active retinal cells [30]. This binding triggers protein aggregation and subsequent proteotoxic stress, leading to mitochondrial respiration collapse [77]. Studies have shown that exposure to copper in hyperglycemic conditions significantly reduces the expression of the mitochondrial fusion protein 2 in retinal pigment epithelial cells [73]. This induces characteristic mitochondrial fragmentation and dysfunction.

In the context of diabetes, hyperglycemia-induced oxidative stress generates reactive oxygen species, which damage copper-binding proteins and release free copper ions. This creates a vicious cycle of metal imbalance and cellular damage [78, 79]. In addition, 4D-DIA quantitative proteomics revealed that the expression of copper transporters (such as ATP7A) is increased in DR mice [80]. This provides evidence of heightened regional copper sensitivity in retinal cells under DR conditions.

### Metabolic regulation of cuproptosis in diabetic cardiovascular complications

The extant clinical evidence indicates an association between systemic copper homeostasis imbalance and adverse cardiovascular outcomes in patients with diabetes. Plasma copper has been identified as an independent risk factor for all-cause mortality in patients with type 2 diabetes (HR = 1.60; 95% CI: 1.30–1.97), exhibiting a J-shaped dose-response relationship [81]. NHANES data further corroborates the finding that dietary copper intake is non-linearly associated with cardiovascular events in high-risk populations, with an optimal intake threshold of approximately 2.85 mg/day, which provides a protective effect [82].

**Fig. 3:**
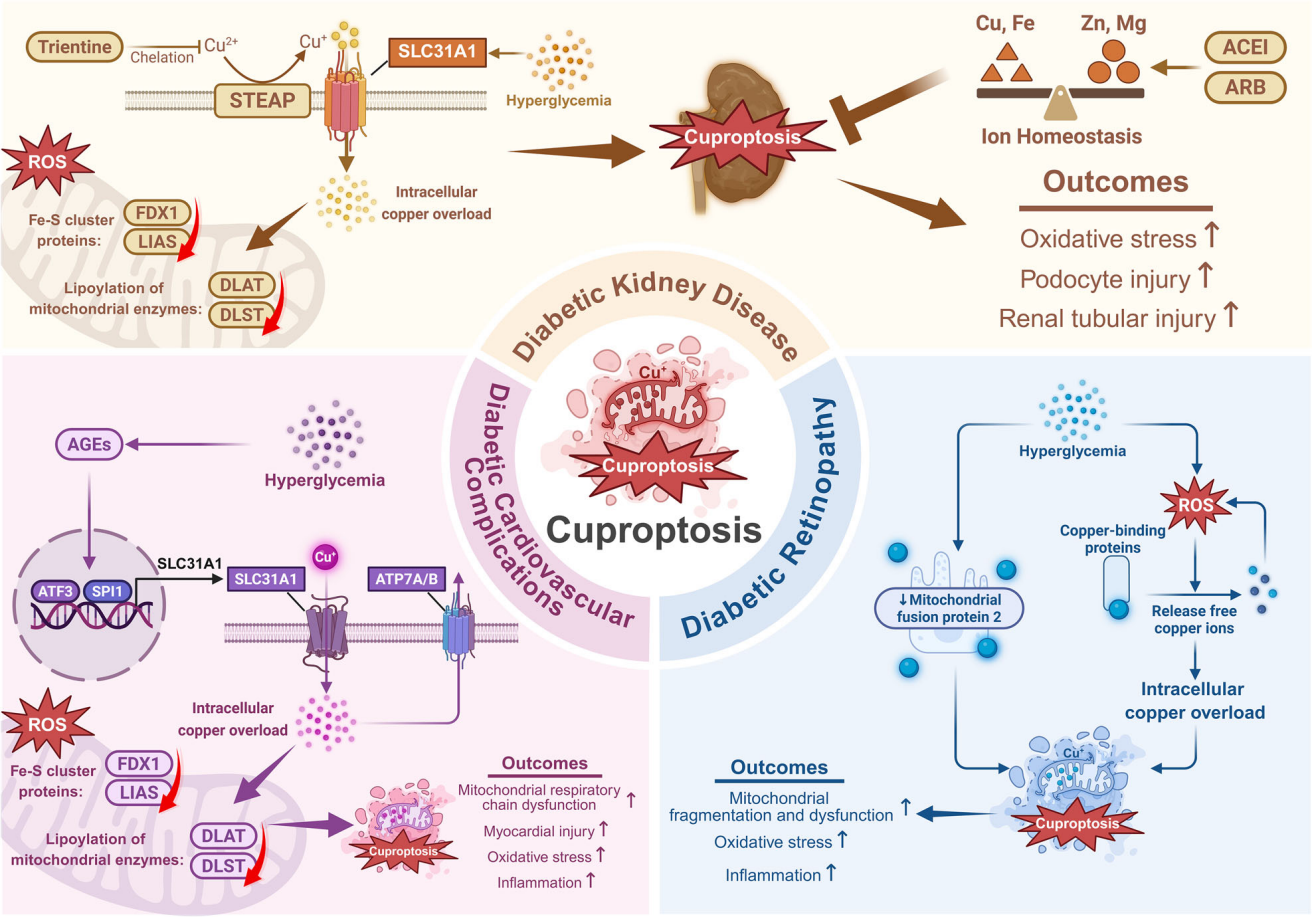
Metabolic dysregulation and cuproptosis in diabetic complications. In Diabetic Kidney Disease (DKD) (top), cuproptosis is characterized by the loss of Fe-S cluster proteins (FDX1, LIAS) and aberrant lipoylation of mitochondrial enzymes (DLAT, DLST). The copper chelator Trientine suppresses this process by sequestering free copper ions, while ACEI and ARB therapies delay disease progression by restoring intracellular ion homeostasis. In Diabetic Retinopathy (DR) (bottom right), hyperglycemia-induced ROS triggers the release of excess free copper from copper-binding proteins. Concurrently, the combined stress of high glucose and copper exposure downregulates mitochondrial fusion protein 2, thereby contributing to cuproptosis. In Diabetic Cardiovascular Complications (DCVC) (bottom left), cuproptosis is promoted by hyperglycemia-induced AGEs, which upregulate the copper transporter SLC31A1 via the transcription factors ATF3 and SPI1. DKD: Diabetic Kidney Disease; Fe-S: Iron-Sulfur; FDX1: Ferredoxin 1; LIAS: Lipoic Acid Synthetase; DLAT: Dihydrolipoamide S-Acetyltransferase; DLST: Dihydrolipoamide S-Succinyltransferase; ACEI: Angiotensin-Converting Enzyme Inhibitor; ARB: Angiotensin II Receptor Blocker; DR: Diabetic Retinopathy; ROS: Reactive Oxygen Species; DCVC: Diabetic Cardiovascular Complications; AGEs: Advanced Glycation End Products; SLC31A1: Solute Carrier Family 31 Member 1; ATF3: Activating Transcription Factor 3; SPI1: Spi-1 Proto-Oncogene.

At the molecular level, the accumulation of advanced glycation end products (AGEs) in conditions of persistent hyperglycemia has been shown to upregulate the transcription of the copper transporter SLC31A1 via the transcription factors ATF3 and SPI1 [83]. This has been demonstrated to lead to intracellular copper overload, the loss of Fe-S cluster proteins (FDX1, LIAS) and reduced lipoylation of mitochondrial enzymes (DLAT, DLST). This pathway positions cuproptosis as a direct mechanistic link between diabetic metabolic insult and myocardial injury. The resulting mitochondrial respiratory chain dysfunction and cardiomyocyte death have been identified as the direct link between metabolic damage in diabetes and myocardial injury (Fig. 3).

## The role of disulfidptosis in diabetes and its complications

### Role of disulfidptosis in diabetic neuropathy

Although there is currently no direct evidence linking disulfidptosis to DN, the metabolic vulnerability of neurons and Schwann cells indicates that this mechanism could be biologically plausible. In cases of diabetes, hyperglycemia inhibits the expression and function of SLC7A11, thereby reducing the uptake of extracellular cysteine by dopaminergic neurons [84]. This results in the depletion of intracellular cysteine, which leads to insufficient GSH synthesis and an imbalance in redox homeostasis. At the same time, the glucose-activated polyol pathway uses up NADPH, which reduces the amount of reducing capacity in the cell [85–87]. These together will lead to the accumulation of cysteine and disulfide bonds, triggering disulfide bond stress.

The polarized structures of neurons and Schwann cells are particularly sensitive to stress in disulfide bonds. Abnormal disulfide bonds can induce the aggregation of cytoskeletal proteins (such as ACTB), which disrupts axonal transport and myelination [88, 89]. Furthermore, the RAC1-WRC pathway amplifies the disulfidptosis cascade and regulates growth cone and Schwann cell regeneration, which could worsen nerve damage [90, 91].

### Role of disulfidptosis in diabetic retinopathy

Key components of disulfidptosis, including the disassembly of the actin cytoskeleton and NADPH oxidase (Nox)-mediated oxidative stress, play a significant role in the pathogenesis of DR.

Hyperglycemia epigenetically activates Rac1, and Rac1 polymorphisms increase the risk of DR in patients with type 2 diabetes [92]. Activation of Rac1 promotes Nox2 assembly, leading to an increase in ROS [93]. Simultaneously, upregulation of Nox4 induces mitochondrial dysfunction and endothelial cell apoptosis [94]. Inhibition of Nox4 significantly improves diabetes-related oxidative damage and vascular permeability, and both factors collectively exacerbate disulfide bond stress and cytoskeletal disruption [95].

Proteomic analysis of the vitreous in patients with proliferative DR has confirmed a significant enrichment of the “actin cytoskeleton regulation” pathway [96]. Disruption of this pathway directly leads to increased permeability of the blood-retinal barrier. Key features of early cytoskeletal remodeling in DR include abnormalities in the regulation of the actin-microtubule network coordinated by MACF1, and pathological remodeling of stress fibers resulting from the transdifferentiation of α-SMA-positive cells, such as myofibroblasts derived from clear cells in the vitreous [97–99]. This remodeling process involves disrupted MACF1 cross-linking and the abnormal accumulation of α-SMA in vascular walls and stromal cells. It directly mediates abnormal retinal vascular perfusion and neovascularization, thereby constituting the core pathological pathway underlying perivascular cell dysfunction, endothelial dysfunction and neurovascular unit failure in DR.

### Research progress of disulfidptosis in diabetic cardiovascular complications

Hyperglycemia has been demonstrated to induce significant activation of thioredoxin-interacting protein (TXNIP), which exerts a “double blow” on redox homeostasis by inhibiting thioredoxin activity and exacerbating endoplasmic reticulum stress [100, 101]. This damage specifically targets the disulfide bond network within adhesion molecules, leading to functional abnormalities in cadherin and integrin heterodimers [102, 103]. This, in turn, triggers the progressive impairment of the endothelial barrier and disruption of myocardial junction integrity, thereby paving the way for arrhythmias [104, 105].

In DCVC, metabolic reprogramming is characterized by a shift towards fatty acid oxidation. Meanwhile, hyperglycemia activates NADPH-consuming pathways and inhibits flux through the pentose phosphate pathway (PPP) [106, 107]. The activation of the aldose reductase pathway and the upregulation of Nox4 further accelerate NADPH depletion [85]. NADPH depletion also impairs the activity of the thioredoxin and glutathione systems, ultimately inducing a reductive crisis that renders cells susceptible to protein aggregation and cytoskeletal instability induced by disulfide bond stress [108].

Strategies that target disulfide bond stress show great promise. Potential interventions include activating Nrf2 to enhance the body’s own antioxidant defenses, inhibiting ERO1α to maintain endothelial function, using small molecules that target cysteine residues in structural proteins and employing metabolic modulators that restore NADPH production and alleviate lipid overload [109–111].

### The potential role of disulfidptosis in diabetic kidney disease

DKD is characterized by metabolic dysfunction and redox imbalance. The progression of disulfidptosis is driven by the interplay of dynamic cytoskeletal dysregulation, defects in the cellular defense network and heightened metabolic susceptibility.

Dynamic cytoskeletal dysregulation is a key driver of disulfide bond-mediated cell death. The downregulation of CKAP4 specifically in DKD directly disrupts the integrity of the actin and microtubule networks, resulting in the disappearance of foot processes [112]. While hyperglycemia-induced upregulation of AEP exerts a protective compensatory effect by cleaving cofilin-1 to produce fragments that stabilize the cytoskeleton, excessive GSK3β activity exacerbates disruption to the actin cytoskeleton in podocytes and increases oxidative stress, further compromising cellular structural stability [113, 114].

This dual damage, caused by structural abnormalities and oxidative stress, interacts synergistically with defects in the antioxidant defense network. The antioxidant protein DsbA-L mitigates oxidative damage by stabilizing peroxidases and maintaining the integrity of the mitochondrial-associated endoplasmic reticulum (MAM) membrane [115]. It also inhibits NLRP3 inflammasome activation [116]. However, its absence weakens the cell’s resistance to disulfidptosis.

**Fig. 4:**
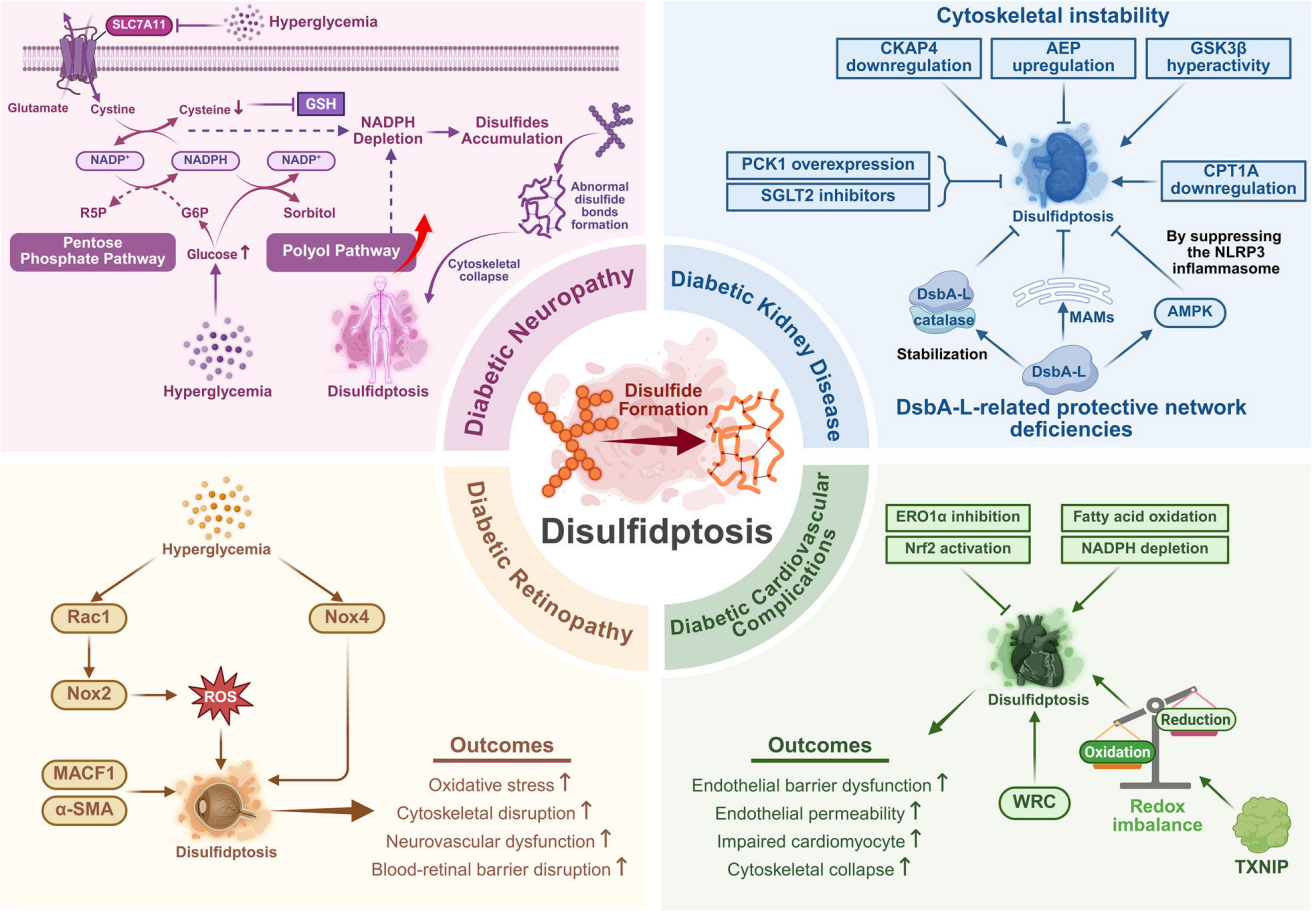
Metabolic dysregulation and disulfidptosis in diabetic complications. In Diabetic Neuropathy (DN) (top left), hyperglycemia drives redox imbalance by diverting glucose through the polyol pathway, depleting NADPH. Simultaneously, hyperglycemia-mediated SLC7A11 downregulation impairs cystine uptake and GSH synthesis. This combined antioxidant depletion triggers catastrophic disulfide bond stress. In Diabetic Kidney Disease (DKD) (top right), disulfidptosis is driven by cytoskeletal instability (regulated by CKAP4, AEP, and GSK3β), lipotoxicity-associated metabolic reprogramming (linked to CPT1A, PCK1, and SGLT2 inhibitors), and a deficient DsbA-L protective network. In Diabetic Retinopathy (DR) (bottom left), hyperglycemia induces excessive ROS via Rac1-mediated Nox2 activation and Nox4 upregulation. This oxidative surge, combined with disrupted MACF1 cross-linking and α-SMA-mediated stress fiber remodeling, accelerates disulfidptosis. In Diabetic Cardiovascular Complications (DCVC) (bottom right), disulfidptosis is promoted by TXNIP-induced redox imbalance, enhanced fatty acid oxidation, NADPH depletion, and WRC. These factors collectively trigger a reductive crisis and cytoskeletal instability. Conversely, Nrf2 activation and ERO1α inhibition protect against disulfidptosis. DN: Diabetic Neuropathy; NADPH: Nicotinamide Adenine Dinucleotide Phosphate; SLC7A11: Solute Carrier Family 7 Member 11; GSH: Glutathione; DKD: Diabetic Kidney Disease; CKAP4: Cytoskeleton Associated Protein 4; AEP: Asparaginyl Endopeptidase; GSK3β: Glycogen Synthase Kinase 3 Beta; CPT1A: Carnitine Palmitoyltransferase 1A; PCK1: Phosphoenolpyruvate Carboxykinase 1; SGLT2: Sodium-Glucose Cotransporter 2; DsbA-L: Disulfide Bond Formation Protein A-Like; DR: Diabetic Retinopathy; ROS: Reactive Oxygen Species; Rac1: Rac Family Small GTPase 1; Nox2: NADPH Oxidase 2; Nox4: NADPH Oxidase 4; MACF1: Microtubule Actin Crosslinking Factor 1; α-SMA: Alpha-Smooth Muscle Actin; DCVC: Diabetic Cardiovascular Complications; TXNIP: Thioredoxin-Interacting Protein; WRC: WAVE Regulatory Complex; Nrf2: Nuclear Factor Erythroid 2-Related Factor 2; ERO1α: Endoplasmic Reticulum Oxidoreductase 1 Alpha.

At the same time, lipotoxicity and metabolic reprogramming increase susceptibility to cellular stress. Downregulation of CPT1A leads to impaired fatty acid oxidation, abnormal lipid accumulation and depleted NADPH reserves, thereby exacerbating cellular metabolic stress [117]. In contrast, overexpression of PCK1 alleviates mitochondrial defects and fibrosis [118]. Furthermore, SGLT2 inhibitors can rescue the downregulation of the splicing regulator Srsf7 induced by diabetes by mimicking a fasting state [119], highlighting the role of metabolic and post-transcriptional regulation in disease progression. However, these compensatory regulatory mechanisms are unable to reverse the overall trend of metabolic imbalance and ultimately contribute to the onset of disulfidptosis (Fig. 4) (Table 2).

**Table 2:** The role of four types of cell death in diabetes and its complications.

Cell Death Type	Major associated diabetes complications	Core-affected cells/tissues	Key Activation Pathways in Complications	Core effects driving complications	References
Pyroptosis	DKD; DR; DCVC; DN	1. DKD: Renal tubular epithelial cells; podocytes, mesangial cells, 2. DR: Retinal endothelial cells, retinal pigment epithelial cells; 3. DCVC: Cardiomyocytes, vascular endothelial cells; 4. DN: Spinal dorsal horn microglia, Schwann cells, epidermal cells	1. DKD: SGLT2-SGK1-NLRP3 axis; 2. DR: P2X7/NLRP3/Caspase-1/GSDMD axis; 3. DCVC: FIS1-DRP1-mtROS-NLRP3 axis, 4. DN: TBK1-noncanonical NF-κB-NLRP3 axis (microglia)	1. DKD: Renal parenchymal cell lysis, release of IL-1β/IL-18, promotion of renal fibrosis; 2. DR: Destruction of retinal neurovascular unit, vascular hyperpermeability, retinal inflammation; 3. DCVC: Myocardial inflammation, mitochondrial fragmentation, vascular endothelial dysfunction; 4. DN: Neuroinflammation, impaired nerve conduction	[39, 41, 44, 46, 47, 49, 51, 53, 54, 56, 58, 60]
Ferroptosis	DN; DCVC; DKD	1. DN: Schwann cells, cardiac autonomic ganglion cells; 2. DCVC: Cardiomyocytes, cardiac microvascular endothelial cells; 3. DKD: Renal tubular epithelial cells	1. DN: AMPK/SIRT1/PGC-1α axis; 2. DCVC: AMPK/NRF2 axis; 3. DKD: AMPK/NRF2 axis	1. DN: Schwann cell dysfunction, impaired myelin synthesis; 2. DCVC: Damage to cardiac microvessels, mitochondrial stress, and a tendency for lipid peroxidation to occur in cardiovascular tissues; 3. DKD: Damage and fibrosis of podocytes in the renal tubules; endoplasmic reticulum stress	[61–65, 68, 69]
Cuproptosis	DKD; DR; DCVC	1. DKD: Renal tubular epithelial cells, glomerular mesangial cells; 2. DR: Retinal pigment epithelial cells, metabolically active retinal cells; 3. DCVC: Cardiomyocytes	1. DKD: Oxidative stress pathway, trace element metabolism disorder pathway; 2. DR: High glucose-copper-induced mitochondrial fusion protein 2 downregulation; ATP7A/CTR1 copper transporter dysregulation 3. DCVC: AGEs-ATF3/SPI1/SLC31A1 axis;	1. DKD: Renal tubular atrophy leads to impaired copper excretion, exacerbating copper accumulation in renal tissue; 2. DR: Mitochondrial respiration collapse in retinal cells, disruption of retinal redox homeostasis, vascular pericyte loss; 3. DCVC: Cardiomyocyte mitochondrial dysfunction, respiratory chain collapse, endothelial dysfunction	[72, 74, 77–80, 83]
Disulfidptosis	DN; DR; DCVC; DKD	1. DN: Neurons, Schwann cells 2. DR: Retinal endothelial cells, retinal ganglion cells 3. DCVC: Cardiomyocytes, vascular endothelial cells 4. DKD: Podocytes, renal tubular epithelial cells	1. DN: SLC7A11-cystine accumulation-disulfide stress axis; 2. DR: Rac1-Nox2/4-ROS-disulfide stress axis; 3. DCVC: TXNIP-thioredoxin inhibition-disulfide stress axis; 4. DKD: CKAP4 downregulation-actin/microtubule disruption axis	1. DN: Neuronal cytoskeleton collapse, Schwann cell myelination disorder; 2. DR: Disruption of blood-retinal barrier, retinal ganglion cell death; 3. DCVC: Endothelial barrier dysfunction, cardiomyocyte adhesion failure; 4. DKD: Podocyte foot process effacement, renal tubular cell structural damage	[84, 88, 90, 96, 97, 100, 106, 109, 112, 113, 115]

## Therapeutic strategies and clinical trials for metabolic cell death

Agents that have entered registered clinical trials (ClinicalTrials.gov identifiers in Table 3) have been explored for their potential to modulate pyroptosis, ferroptosis, and disulfidptosis and related metabolic disorders based on preclinical evidence. For pyroptosis, melatonin, hydrogen combined with metformin, and sodium butyrate plus inulin have been investigated, primarily targeting inflammasome-mediated signaling pathways including AMPK/mTOR and TLR2/4–NF-κB, as well as downstream caspase-1 and GSDMD-mediated pore formation [120–122]. In ferroptosis, SGLT2 inhibitors such as empagliflozin, dapagliflozin, and canagliflozin have been tested for their roles in mitigating iron-dependent lipid peroxidation and oxidative stress, largely through activation of AMPK and NRF2 pathways and reinforcement of system Xc − /GSH/GPX4 defenses [68, 123, 124]. The GLP-1 receptor agonist semaglutide has similarly been evaluated for antioxidant and mitochondrial-protective effects relevant to ferroptotic processes [125]. Nutraceuticals with antioxidant properties, including curcumin, quercetin, and sulforaphane, have been studied for their ability to enhance endogenous redox defenses and suppress lipid peroxidation in diabetic tissues [64, 69, 126]. Although research into drugs that specifically target cuproptosis is limited, it is possible to indirectly inhibit this process by chelating free copper. Trientine tetrahydrochloride, a widely used copper chelating agent in the treatment of Wilson’s disease, has been shown in clinical trials to improve left ventricular hypertrophy in patients with type 2 diabetes [127, 128]. This could offer a new therapeutic approach for inhibiting cuproptosis in diabetes. For disulfidptosis, FGF21-based therapies may modulate iron homeostasis and mitochondrial function, while N-acetyl-cysteine has been explored as a redox and disulfide stress regulator [108, 129]. Collectively, these interventions illustrate the current translational landscape for targeting metabolism-linked programmed cell death, offering potential combinatorial strategies to attenuate multi-organ injury in diabetes while bridging mechanistic insights from preclinical studies to human clinical evaluation (Table 3).

**Table 3:** Therapeutic Drugs for Metabolic Cell Death.

Drug	Mechanism	Target	Cell Death Type	Treatment Time	Drug Dosage	Clinical Trial ID	References
Melatonin	Inhibiting pyroptosis by upregulating miR-214-3p to reduce caspase-1 protein levels	miR-214-3p	Pyroptosis	8 weeks	10 mg/kg/day	NCT07036796	[120]
Hydrogen + Metformin	Inhibiting pyroptosis by inhibiting the NLRP3 inflammasome via the AMPK/mTOR signaling pathway	AMPK	Pyroptosis	8 weeks	200 mg/kg/day metformin+inhaled 2% hydrogen for 3 h per day	NCT01690091	[121]
Sodium butyrate + inulin	Inhibiting NLRP3/caspase-1-mediated pyroptosis by upregulating miR-146a-5p and miR-9-5p to inhibit the TLR2/4-NF-κB signaling pathway	miR-146a-5p and miR-9-5p	Pyroptosis	45 days	600 mg/day sodium butyrate+10 g/day inulin	NCT07252609	[122]
Dapagliflozin	Inhibiting ferroptosis by suppressing the HIF1α/HO1 axis, thereby reducing iron overload and lipid peroxidation	SGLT2	Ferroptosis	13 weeks	1 mg/kg/day	NCT02682563	[123]
Canagliflozin	Inhibiting ferroptosis by balancing cardiac iron homeostasis and promoting the system Xc-/GSH/GPX4 axis	SGLT2	Ferroptosis	6 weeks	20 mg/kg/day	NCT01032629	[124]
Semaglutide	Inhibiting ferroptosis by enhancing antioxidant defenses via β-Klotho–AMPK signaling	GLP-1R	Ferroptosis	twice a week, 8 weeks	60 μg/kg	NCT04865770	[125]
Curcumin	Inducing PKCδ-mediated p62 phosphorylation, leading to NRF2 activation, thereby enhancing cellular antioxidant capacity and inhibiting ferroptosis	PKCδ	Ferroptosis	daily or every other day for 3 months	300 mg/kg/day	NCT03262363	[126]
Quercetin	Activating the Nrf2/HO-1 pathway, which upregulates GPX4 and SLC7A11 and downregulates TFR-1	NRF2	Ferroptosis	five times per week for 12 weeks	25 mg/kg (low dose) or 100 mg/kg(high dose)	NCT01839344	[69]
N-acetyl-cysteine	Inhibiting disulfidptosis by reducing accumulated abnormal disulphide bonds, thereby preventing cytoskeletal protein crosslinking	Accumulated abnormal disulfide bonds	Disulfidptosis	3 weeks	10 mg/kg	NCT00556465	[108]
Trientine tetrahydrochloride	Selective chelation of Cu^2+^ to promote urinary Cu excretion; normalize copper homeostasis disrupted by hyperglycemia; ameliorate left ventricular hypertrophy without altering blood glucose or blood pressure; suppress pathogenic TGF-β activation	Cu^2+^; copper homeostasis-related pathways; TGF-β signaling pathway	Cuproptosis	12 months	600 mg orally twice daily	NCT01213888	[127]

## Interaction between cell death modalities and comorbidity mechanisms

### Oxidative-inflammatory synergistic amplification between pyroptosis and ferroptosis

Abnormal glucose metabolism in diabetes induces mitochondrial dysfunction, resulting in the excessive production of ROS, which simultaneously drives inflammasome-mediated pyroptosis and ferroptosis, providing the molecular basis for the two processes to interact synergistically [130]. The activation of pyroptosis releases IL-1β and IL-18. IL-1β can upregulate NF-κB by recruiting MyD88, which may suppress GPX4 expression through inhibition of the SLC7A11-dependent antioxidant system [131, 132]. This impairs the capacity to clear lipid peroxides. Furthermore, the accumulation of lipid ROS during non-canonical pyroptosis may represent a late event that contributes to ferroptosis [133].

In ferroptosis, lipid peroxidation promotes pyroptosis by engaging multiple key nodes within the pyroptotic pathways, including the activation of the caspase-11 non-canonical pathway, mitochondrial damage-mediated NLRP3 inflammasome activation, and GSDMD-N membrane localization and pore formation via PLCγ1-Ca²⁺ signaling [134]. Interestingly, emerging evidence suggests that ALOX5 and the cGAS–STING pathway may promote both pyroptosis and ferroptosis [133, 135]. However, it remains to be elucidated whether they act as common upstream signals or whether they serve as mediators of direct crosstalk only when one cell death modality sequentially triggers the other.

### Metal ion metabolism coupling between cuproptosis and ferroptosis

Diabetes-associated hyperglycemia disrupts copper–iron homeostasis, and because these metals are tightly interconnected, such an imbalance may couple cuproptosis and ferroptosis through shared redox and mitochondrial vulnerabilities [136]. Systemically, copper-dependent multicopper ferroxidases (hephaestin and ceruloplasmin) link copper status to iron export and distribution [137]. Copper deficiency reduces their activity, while dual loss leads to local tissue iron overload despite systemic iron deficiency, a condition that may intensify iron-driven oxidative stress in specific organs [138]. At the execution level, ferroptosis is restrained by the GSH–GPX4 axis, whereas cuproptosis sensitivity is likewise influenced by cellular thiol/redox capacity [139]. Notably, copper can promote ferroptosis via macroautophagy-dependent GPX4 degradation, establishing a direct Cu to GPX4 connection between the two pathways [140]. Conversely, copper-ionophore models (e.g., DSF/Cu) exhibit concurrent features of both death modes, including GSH depletion, lipid peroxidation, DLAT aggregation, and Fe–S protein loss, highlighting GSH as a shared regulatory node [141]. This convergence extends upstream to NRF2 signaling, which governs both iron metabolism and GSH synthesis [142]. In addition, mitochondrial dysfunction provides a central intersection, where copper-induced disruption of Fe–S proteins and increased ROS may amplify lipid peroxidation, thereby linking cuproptosis with ferroptosis in diabetic contexts [83, 143]. These observations collectively suggest that copper and iron metabolism are not parallel regulators but components of a unified redox–mitochondrial network that integrates cuproptosis and ferroptosis.

### Inflammation-metabolism reprogramming between pyroptosis and cuproptosis

In diabetes and its complications, cuproptosis and pyroptosis should be viewed as mechanistically convergent rather than fully independent processes, because recent articles place them within the same copper–mitochondria–inflammation axis. In diabetic myocardium, AGEs activate the ATF3/SPI1/SLC31A1 pathway, increase copper uptake, and produce a cuproptosis-associated injury pattern, indicating that hyperglycemia can establish a copper-loading state in diabetic tissue [83]. In diabetic retinopathy, the STAT1/SLC31A1 axis likewise promotes cuproptosis and is accompanied by microglial activation and increased pro-inflammatory cytokine expression, linking a cuproptosis-related program to retinal inflammation in a disease-relevant setting [144]. In parallel, pyroptosis has been directly demonstrated in diabetic retina and diabetic cardiomyopathy through P2X7/NLRP3- and mtDNA–cGAS–STING–NLRP3-dependent signaling, respectively [46, 54]. A key bridge is that copper overload itself can trigger mtDNA leakage, cGAS–STING activation, NLRP3 assembly, and pyroptosis, supporting the idea that diabetic copper dyshomeostasis can feed directly into inflammasome-driven cell death [145]. Taken together, cuproptosis-related copper dysregulation and pyroptosis-related inflammasome activation are mechanistically convergent processes that likely reinforce one another through mitochondrial stress and inflammatory amplification.

### The redox hub role of disulfidptosis

Disulfidptosis is a novel form of cell death that interacts with pyroptosis, ferroptosis and cuproptosis in complex ways. These processes collectively contribute to the progression of diabetes and its complications.

Under high-glucose conditions, pyroptosis and disulfidptosis interact closely via redox dysregulation, which is primarily driven by ROS. NADPH is the cornerstone of the cellular antioxidant system, and its insufficiency directly impairs cellular antioxidant capacity, for example, by reducing the function of the GSH/GPX4 system [146]. This leads to a significant increase in ROS levels. ROS act as a key upstream signal that effectively activates the NLRP3 inflammasome, thereby initiating the pyroptosis pathway [147]. This creates a cycle of synergistic damage involving ‘disulfidptosis–ROS–pyroptosis’, which collectively worsens the pathological damage caused by high glucose levels. The amplifying effect of pyroptosis interacts with the structural damage to cells caused by disulfidptosis. Together, they form a synergistic damage network driven by redox imbalance in the pathological microenvironment of diabetes.

In DKD, the synergistic effects of disulfidptosis and ferroptosis, which are caused by redox imbalance and metabolic disruption, exacerbate renal tubular cell death [148]. Hyperglycemia-induced oxidative stress can trigger GSH depletion. Meanwhile, oxidative stress-mediated damage to organelle membranes leads to the release and overload of iron, which are essential conditions for ferroptosis [149]. In a high-glucose environment, the downregulation of SLC7A11 expression and insufficient cysteine uptake reduce GSH synthesis [150]. This can indirectly promote disulfide bond stress through redox imbalance, creating a vicious cycle of synergistic damage from both disulfidptosis and ferroptosis.

Both cuproptosis and disulfidptosis are closely associated with imbalances in cellular redox homeostasis, and they can amplify each other’s effects through NADPH depletion. Cell experiments have demonstrated that treatment with the cuproptosis inducer Es/Cu (a complex of Elesclomol and CuCl₂) results in a significant decrease in NADPH levels in HeLa cells [151]. This change is driven by Fe-S cluster depletion and ROS-mediated oxidation. Depletion of NADPH disrupts the balance of thiol-disulfide bonds in cells, triggering abnormal disulfide cross-linking in the actin cytoskeleton. This ultimately leads to cytoskeletal collapse and disulfidptosis [36].

### Targeting interactive networks for synergistic therapeutic strategies

**Fig. 5:**
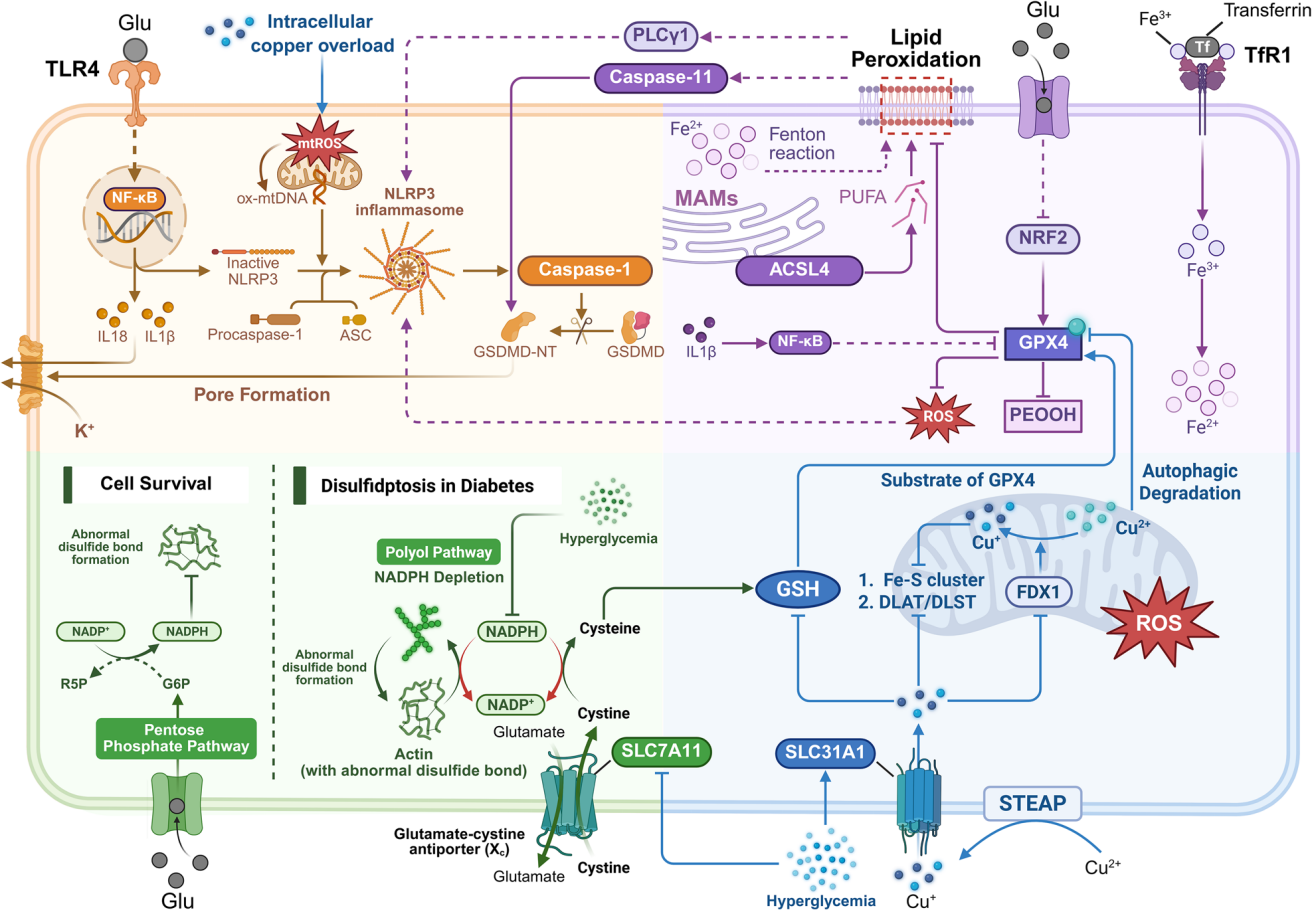
Molecular crosstalk among pyroptosis, ferroptosis, cuproptosis and disulfidptosis in Diabetic Complications. **A** Interconnected network of pyroptosis, ferroptosis, and cuproptosis. Pyroptotic signaling cascades may promote ferroptosis by enhancing oxidative stress and impairing GPX4 activity. Ferroptosis and cuproptosis can, in turn, activate both canonical and non-canonical pyroptotic pathways at multiple regulatory nodes. Cuproptosis may interact with ferroptosis through mitochondrial dysfunction and disruption of the GSH/GPX4 antioxidant system, thereby promoting lipid peroxidation. **B** Disulfidptosis can function as a bidirectional redox hub. Driven by hyperglycemia itself, ferroptosis-associated GSH depletion, and cuproptosis-induced Fe-S disruption, NADPH depletion contributes to abnormal disulfide bond formation and cytoskeletal collapse. Concurrently, this reductive crisis promotes ROS accumulation, forming a feed-forward loop that may exacerbate inflammasome-mediated pyroptosis, cuproptosis-associated proteotoxic stress, and iron-dependent lipid peroxidation. GPX4: Glutathione Peroxidase 4; GSH: Glutathione; Fe-S: Iron-Sulfur; NADPH: Nicotinamide Adenine Dinucleotide Phosphate; ROS: Reactive Oxygen Species.

Due to the intricate interactions among the four programmed cell death pathways outlined above, targeting the key regulatory nodes that are common to multiple pathways can lead to the synergistic inhibition of various forms of cell death. This approach can overcome the limitations of interventions that target a single pathway. GSH is a key cofactor in the antioxidant function of GPX4. In the renal tubular cells of patients with DKD, GSH levels are significantly reduced [68]. This is accompanied by the downregulation of GPX4 expression and the accumulation of lipid peroxides (such as MDA and 4-HNE), which ultimately triggers ferroptosis. In addition, the sulfhydryl group (-SH) of GSH has a high affinity for Cu⁺, allowing it to be transported into mitochondria via the mitochondrial transporter SLC25A39 [139]. There, it forms a stable GSH-Cu⁺ complex with free Cu⁺. This prevents toxic stress to mitochondrial proteins and inhibits cuproptosis. Crucially, the mitochondrial-associated endoplasmic reticulum membrane (MAM) acts as a key hub for signaling between organelles and controls both pyroptosis and ferroptosis. It provides a physical platform for the assembly of the NLRP3 inflammasome [152]. NLRP3 and its adaptor ASC colocalize in the MAM region to form a functional complex that mediates pyroptosis. At the same time, the MAM regulates iron transport via the ZIP7-VDAC3 axis [153]. ZIP7, which is located on the endoplasmic reticulum membrane, interacts with VDAC3, which is located on the outer mitochondrial membrane, to mediate the transport of iron from the endoplasmic reticulum to the mitochondria. Meanwhile, ACSL4, which is localized to the MAM, binds to ZIP7 and VDAC3, thereby promoting iron transport by modulating their interaction. Furthermore, cuproptosis leads to the downregulation of Fe-S cluster proteins, such as FDX1 and LIAS [154]. This, in turn, induces the accumulation of ROS derived from the MAM and mitochondria. Since ROS are a key trigger for NLRP3 inflammasome activation, this ultimately drives pyroptosis. Therefore, targeting functional molecules in the MAM region, such as VDAC1, ACSL4 and ZIP7, can block both the pyroptosis and ferroptosis pathways, as well as interfering with the interaction between cuproptosis and pyroptosis. This offers a novel therapeutic approach to pathological conditions involving synergistic damage caused by multiple types of cell death. Examples include diabetes-related complications such as severe diabetic limb ischemia (Fig. 5).

## Conclusions and future prospects

Although a growing body of research indicates a link between different cell death pathways and diabetic complications, clinical investigations into how interactions among these pathways exacerbate diabetes-related tissue damage are scarce. This review explores the interplay between multiple cell death pathways in the context of diabetic complications. Understanding these interaction networks is essential for creating effective therapeutic strategies. Current clinical interventions for diabetes-related complications largely focus on regulating a single pathway when targeting metabolic cell death. However, as these four modes of cell death are part of the same core tripartite pathway involving “oxidative stress–inflammation–metal ions”, targeting a single pathway with monotherapy is often insufficient to achieve lasting therapeutic effects. This can even trigger the activation of other death pathways, resulting in incomplete suppression of pathological damage. By contrast, multi-target intervention strategies that focus on core regulatory nodes shared by several cell death pathways (e.g., JNK1, GSH/GPX4 and MAMs) are more likely to disrupt the synergistic amplification loops of cell death. This makes them a more effective and comprehensive way of mitigating multi-organ damage in diabetes.

It is worth noting that the core pathological features of diabetes form a synergistic network comprising chronic hyperglycemia, persistent oxidative stress, inflammatory activation and imbalance in metal ion homeostasis. This unique microenvironment simultaneously amplifies the activation efficiency of all four modes of cell death. Furthermore, key target cells for diabetic complications, such as renal podocytes, retinal endothelial cells and cardiomyocytes, exhibit significantly higher sensitivity to these four modes of cell death than other cells, due to their specific metabolic characteristics (e.g., retinal cells have high copper transport requirements, cardiomyocytes have high mitochondrial content and podocytes have high redox dependence). This ‘selective sensitivity’ results in these four cell death pathways having a more pronounced pathological contribution in diabetic target organs. Future research should utilize technologies such as multi-omics analysis, single-cell sequencing and in vivo animal models to identify organ- and cell-specific core regulatory nodes. Concurrently, it is essential to develop molecular biomarkers that can detect the activation of various metabolic cell death pathways, and to establish treatment strategies that are tailored to each patient’s individual biomarker profile. This will provide new therapeutic options and ultimately improve the prognosis and quality of life for patients with diabetes and its complications.
